# Comparison of warm sitz bath and electronic bidet with a lower-force water flow for postoperative management after hemorrhoidectomy (BIDLOW)

**DOI:** 10.1186/s12893-024-02737-0

**Published:** 2025-01-06

**Authors:** Yoon-Hye Kwon, Seung-Bum Ryoo, Heung-Kwon Oh, Jae Bum Lee, Hyung-Joong Jung, Kee-Ho Song, Seung Chul Heo, Rumi Shin, Joongyub Lee, Kyu Joo Park

**Affiliations:** 1 Department of Surgery, Seoul National University Hospital, Seoul National University College of Medicine, Seoul, Korea; 2 Department of Surgery, Uijeongbu Eulji Medical Center, Uijeongbu City, Korea; 3 Department of Surgery, Seoul National University Bundang Hospital, Seoul National University College of Medicine, Seongnam, Korea; 4 Department of Surgery, Daehang Hospital, Seoul, Korea; 5 Department of Surgery, Goodjang Hospital, Seoul, Korea; 6 Department of Surgery, Seoul National University Boramae Hospital, Seoul National University College of Medicine, Seoul, Korea; 7 Division of Clinical Epidemiology, Biomedical Research Institution, Medical Research Collaborating Center, Seoul National University Hospital, Seoul, Korea; 8 Division of Colorectal Surgery, Department of Surgery, Seoul National University College of Medicine, Seoul National University Hospital, 101 Daehak-ro (28 Yongon-dong), Jongro-gu, Seoul, 03080 Korea

**Keywords:** Electronic bidet, Hemorrhoidectomy, Pain, Sitz bath, Wound

## Abstract

**Aim:**

Electronic bidets can be a substitute for sitz baths, but no study has examined the use of electronic bidets to manage anal problems.

**Methods:**

A randomized, controlled, single-blind, multicenter, parallel group trial was performed. Patients who underwent hemorrhoidectomy were randomly assigned (1:1) to use the electronic bidet or warm sitz baths for 7 days after hemorrhoidectomy. The primary endpoint was the difference in the anal pain VAS score for 7 days posthemorrhoidectomy.

**Results:**

Patients were assigned to the electronic bidet (51) or sitz bath (50) groups. Twenty-six patients dropped out after randomization, and the final analysis included 34 patients in the electronic bidet group and 41 in the sitz bath group. The VAS score for anal pain 7 days posthemorrhoidectomy did not differ between the electronic bidet and sitz bath groups (38.3 ± 21.9 vs. 42.0 ± 21.1, *p* = 0.453). The upper limit of the 95% confidence interval of the VAS score in the electronic bidet group (81.22) was greater than the margin of noninferiority (46.20).

**Conclusion:**

The VAS scores after hemorrhoidectomy did not differ between the electronic bidet and sitz bath groups, but the noninferiority of the electronic bidet to sitz baths for anal pain 7 days posthemorrhoidectomy was not verified.

**Trial Registration:**

The trial was registered on ClinicalTrials.gov (Registration number: NCT02353156, date: 02/02/2015).

**Supplementary Information:**

The online version contains supplementary material available at 10.1186/s12893-024-02737-0.

## Introduction

Hemorrhoids are the most common anal problem, and hemorrhoidectomy is the ideal treatment for patients who do not respond to conservative management with dietary modification or fiber intake [1, 2]. Open and closed hemorrhoidectomies with excision of prolapsed or engorged hemorrhoid piles have been the standard procedures for treating grade III and IV hemorrhoids. However, some postoperative problems can develop, including bleeding, urinary difficulty, pain or delayed wound healing [3, 4]. Closed hemorrhoidectomy has been considered beneficial in terms of faster wound healing than open hemorrhoidectomy, but healing still takes approximately 3–4 weeks [5, 6]. Although recent advanced techniques, recurrences and unusual complications can still occur [7–9]. Several international guidelines provide thorough recommendations for the management of hemorrhoidal disease The American Society of Colon and Rectal Surgeons (ASCRS) recommends a staged treatment approach utilizing multimodal pain strategies. These include preoperative counseling, regional anesthesia, pharmacological treatments, and non-pharmacological methods such as sitz baths [10]. Similarly, the European Society of Coloproctology (ESCP) guidelines emphasize personalized treatment, focusing on individualized pain assessment, combined pharmacological approaches, patient education, and minimally invasive surgical techniques to reduce postoperative pain [11]. However, these recent guidelines do not provide detailed recommendations for postoperative pain management.

Warm sitz baths are helpful for managing postoperative problems after hemorrhoidectomy [12–14]. Warm-water sitz baths can relieve anal discomfort by reducing anal sphincter spasms to promote tissue healing by increasing blood flow [15, 16]. In previous studies, we have also suggested that warm water can relax the anal sphincter and decrease the anal resting pressure [17, 18]. Although some clinical studies have suggested controversies, many surgeons still recommend warm sitz baths to their patients after anal surgery [19–22]. 

Electronic bidets can be a suitable and convenient substitute for warm sitz baths, and we verified that these automatic devices could reduce anal sphincter pressure, especially when very low-force warm water jets are used [17, 18]. However, there has been no study of the use of electronic bidets to manage postoperative problems after anal surgery.

We aimed to verify the efficacy and safety of our electronic bidet with a lower-force fountain-type water flow system for anal pain control and wound cleansing after hemorrhoidectomy compared to the conventional warm sitz bath.

## Methods

### Patients

This randomized, controlled, single-blind, multicenter, parallel group trial was performed to compare the efficacy and safety of an electronic bidet with a lower-force fountain-type water flow system for anal pain control and wound cleansing after hemorrhoidectomy with those of the conventional warm sitz bath. Patients who required hemorrhoidectomy were eligible for inclusion in this study. The inclusion criteria were adult patients aged 19–70 years who needed hemorrhoidectomy because of hemorrhoid bleeding or protrusion. Exclusion criteria included a history of previous anal surgery, anal abscess or fistula, Crohn’s disease, pregnancy or breast feeding, history of treatment for malignancy within 5 years, immunosuppressant or steroid use, human immunodeficiency virus infection, drug allergy, or any uncontrolled medical problems. All included patients provided written informed consent. The study was conducted in compliance with the Declaration of Helsinki and approved by the institutional review board (IRB) of Seoul National University Hospital (1412-080-633). The trial is registered on ClinicalTrials.gov (NCT02353156, date: 02/02/2015).

### Randomization and masking

The patients were randomly assigned 1:1 before hemorrhoidectomy to either the electronic bidet or sitz bath (control group) for anal pain control and wound cleansing after hemorrhoidectomy using a block permutation approach. The block size was 4 or 6. Random numbers were computer generated by SAS 9.4 (SAS Institute, Cary, NC, USA). Allocation was performed through the Interactive Web Randomization System (IWRS) of the Medical Research Collaborating Center (MRCC) of Seoul National University Hospital by a researcher who was independent from the progress of the study. Throughout the entire study period, the surgeon remained blinded to the treatment the participants received.

### Procedures

Conventional closed or semiclosed hemorrhoidectomy was performed for all patients, and seven surgeons from four hospitals participated in this study. The anoderm and mucosa around the hemorrhoid pile were incised, and careful dissection between the submucosa and internal sphincter muscle was performed with meticulous hemostasis. The main protruding piles were removed completely. The hemorrhoidal artery and pedicle were sutured, and the wound was closed with absorbable suture materials. Preoperative mechanical bowel preparation was recommended 1 day before the operation, and perioperative antibiotics were used immediately before the operation and 1 day after the operation. The patients resumed an oral diet 1 day after the operation. Thirty milligrams of ketorolac tromethamine, a nonsteroidal anti-inflammatory drug (NSAID), was administered by intravenous injection on the operation day and 1 or 2 days after the operation during hospitalization to relieve postoperative anal pain. Intravenous injections could be administered 3 times a day for a total amount of no more than 90 mg. After discharge from the hospital, the patients could take 10 mg ketorolac tromethamine tablets tid or qid orally for 6 days after the operation. The total amount of oral medication used was determined at 1 week after the operation.

**Fig. 1:**
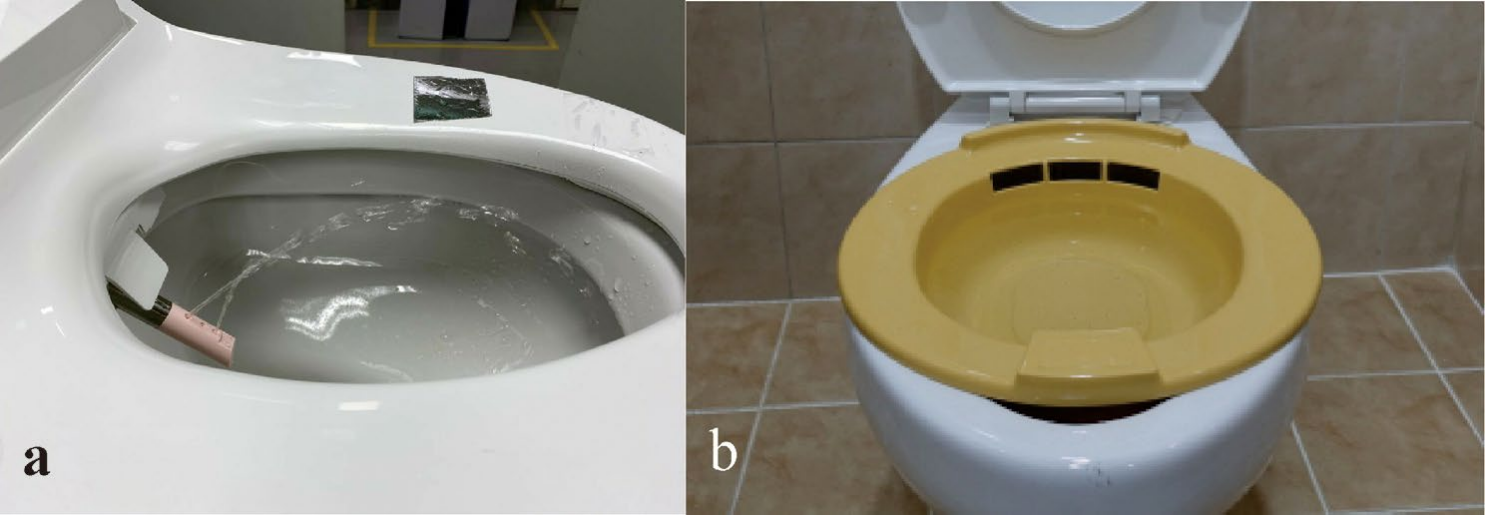
Electronic bidets (**a**) or warm sitz baths (**b**) were used for anal pain control and wound management after hemorrhoidectomy

Electronic bidets or warm sitz baths were used for anal pain control and wound management after hemorrhoidectomy (Fig. 1). We developed an electronic bidet with a fountain-type water flow system that produced a lower-force water stream than commercially available electronic bidets to replace warm sitz baths (Clinic Bidet^®^, Coway Co., Seoul, Korea). The patients in the electronic bidet group applied the water flow of the electronic bidet around the anal wound, and the patients in the sitz bath group applied warm water around their buttocks by sitting in a small tub. The water used in the electronic bidet and sitz bath was warm tap water at approximately 38 °C. The patients were directed to use the electronic bidet or sitz bath for 3–5 min every morning for 7 days after hemorrhoidectomy. The patients could also use their allocated device several times in the daytime when they wanted to alleviate anal pain or clean the perianal wound area for 4 weeks after the operation.

### Efficacy and safety assessment

The severity of anal pain after hemorrhoidectomy was assessed by using the visual analog scale (VAS) (Figure A1). It has been used as a common measurement tool to evaluate subjective characteristics or to quantify pain. The patients recorded their VAS score before and after the electronic bidet or sitz bath every morning for 7 days after hemorrhoidectomy. The primary endpoint was the difference in the VAS score for anal pain after the use of an electronic bidet or sitz bath at 7 days after the operation to assess the efficacy of the electronic bidet for anal pain control after hemorrhoidectomy. Adherence to the procedure was assessed by checking the complete recordings of the VAS score for 7 days. The secondary endpoint was the difference in the wound healing rate at 4 weeks after the operation and the results of a comprehensive patient-reported assessment of the convenience of postoperative care, which was scored as follows: 1: completely inconvenient, 2: considerably inconvenient, 3: neutral, 4: considerably convenient, and 5: completely convenient.

Safety was assessed by the independent data monitoring committee, who monitored adverse events during the study periods. Safety evaluations included a comprehensive analysis of reported adverse events and serious adverse events. Adverse events were classified as definitely, probably, possibly, or remotely related to the interventional procedures.

### Study design

We hypothesized that for the alleviation of anal pain, the electronic bidet would not be inferior to the sitz bath at 7 days after hemorrhoidectomy. Therefore, we planned a noninferiority test with a 10% difference margin, 80% power and 2.5% type I error. The effect of the sitz bath was based on the study of Gupta [21], in which the authors reported an average VAS score of 22.0 (SD: 3.2) for the sitz bath group. The test statistic used was two-sided Fisher’s exact test. The required number calculated for each group was 34.

### Statistical analysis

Baseline characteristics are presented as the means ± standard deviations (SD) for continuous variables and as numbers and percentages for categorical variables. A two-sided 95% confidence interval for the VAS score difference between the treatment groups was evaluated; whether the upper limit of the 95% confidence interval fell within the predetermined margin of noninferiority was checked to provide evidence of the noninferiority of electronic bidets to sitz baths. The conclusion regarding the primary outcome was based on the result from the full analysis set (FAS) population; the per-protocol (PP) analysis result was also provided. The FAS population consisted of patients who used the intervention at least once, while the PP population consisted of patients with more than 80% complete use of the electronic bidet or sitz bath according to the protocol for 7 days. Student’s t test was used to analyze the main outcome: the difference in VAS scores for anal pain. The difference in the wound healing rate at 4 weeks after the operation between the two groups was compared using the chi-square test or Fisher’s exact test, as appropriate. Statistical analysis was performed using SPSS for Windows, version 22.0 (SPSS, Chicago, IL, USA), and statistical significance was assumed for p values < 0.05.

## Results

### Overall study population

**Fig. 2:**
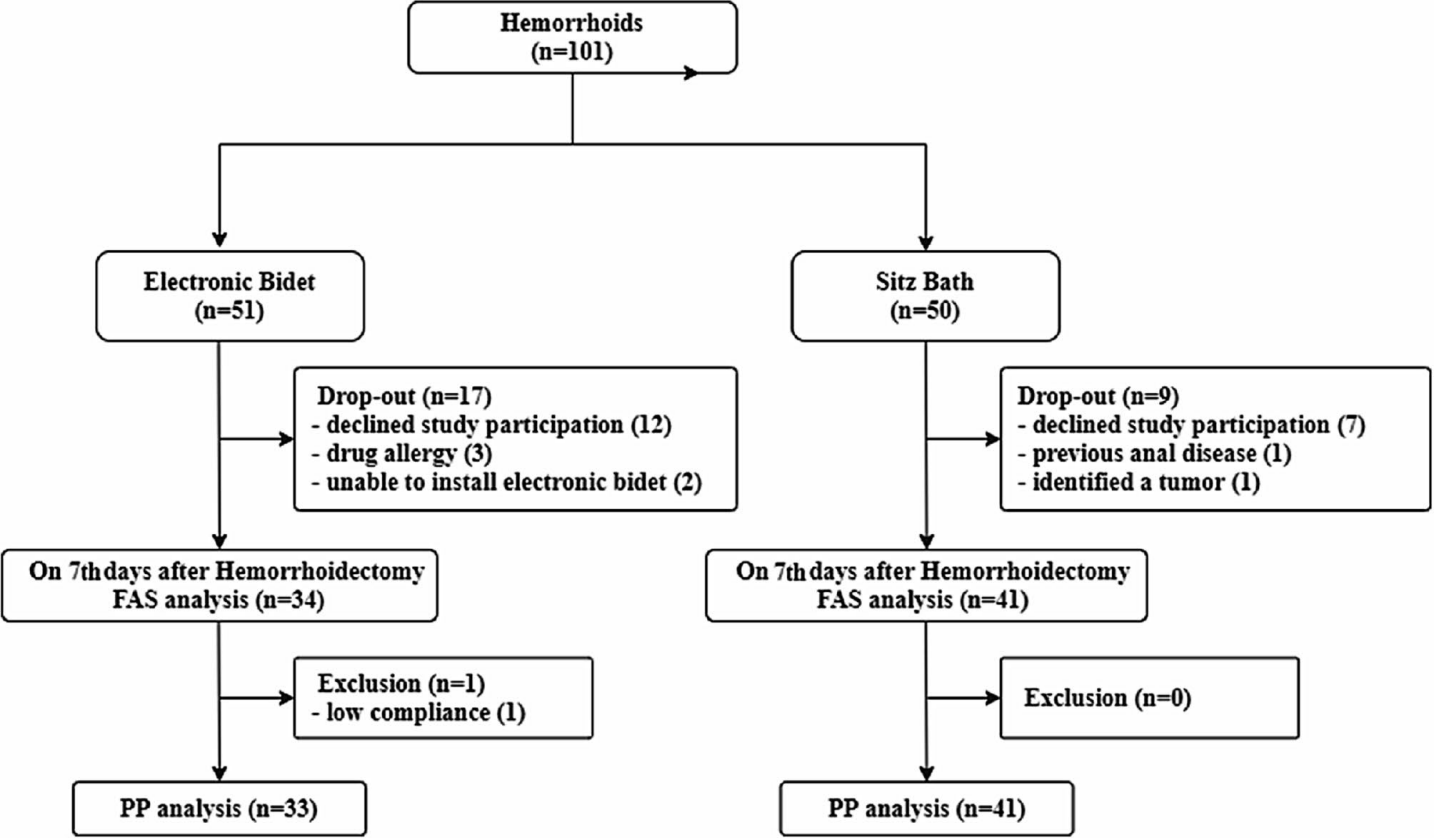
The overall study population and consort diagram

One hundred one patients from 4 hospitals were screened between October 2015 and January 2018 for this prospective, randomized, multicenter study. Among the 101 patients, 51 were assigned to the electronic bidet group, and 50 were assigned to the sitz bath group. Twelve patients in the electronic bidet group and 7 patients in the sitz bath group were excluded because they withdrew from the study after randomization. Three patients in the electronic bidet group presented possible allergies to ketorolac tromethamine after randomization. In the sitz bath group, 1 patient was excluded because of an uncertain previous history of injection therapy for anal disease, and another patient was excluded because a neurogenic pelvic tumor was found before surgery. Two patients in the electronic bidet group who were unable to install electronic bidets at their home were excluded. Thus, the analysis was performed with data from 34 patients in the electronic bidet group and 41 patients in the sitz bath group in the FAS population. Only 1 patient presented below 80% compliance with the intervention in the electronic bidet group and was excluded from the PP analysis. The mean patient age was 50.85 ± 11.35 years (range, 20–68), and 36 patients (48.0%) were male. The overall study population and consort diagram are described in Fig. 2. The demographics and baseline characteristics did not differ significantly between the groups (Table 1). The total amount of ketorolac tromethamine used for pain control did not differ between the electronic bidet and sitz bath groups (143.3 ± 31.0 vs. 220.0 ± 50.8 mg, *p* = 0.214). Additional medications for pain control were necessary for 7 (20.6%) patients in the electronic bidet group and 3 (7.3%) patients in the sitz bath group (*p* = 0.092). Opioids, such as tramadol, were used in 2 patients in the electronic bidet group and 2 patients in the sitz bath group. The others used acetaminophen or another NSAID. The compliance rates for the complete use of the intervention for 7 days were 94.1% (32/34) and 97.6% (40/41) in the electronic bidet group and the sitz bath group, respectively (*p* = 0.449). One patient could not use the electronic bidet on the 4th and 5th days after the operation due to an emergency room visit for severe headache resulting from lumbar puncture for spinal anesthesia; thus, 71.4% of the patients received the intervention. When an analysis of the PP population was performed by using data from 33 patients in the electronic bidet group and 41 patients in the sitz bath group, the results were similar to those of the FAS population (Table A1).

**Table 1:** Demographics and baseline characteristics of the patients

	Bidet (*n* = 34)		Sitz bath (*n* = 41)		*p* value
	*N*	%	*N*	%	
Age, years (mean ± SD)	48.7 (± 11.6)		52.6 (± 11.0)		0.137
Sex, n (%)					
Female	16	47.06	23	56.1	0.435
Male	18	52.94	18	43.9	
Hemorrhoid grade					
II	1	2.94	2	4.88	0.398*
III	31	91.18	39	95.12	
IV	2	5.88	0	0	
Hospitals, n (%)					
1 (SNUH)	5	14.71	13	31.71	0.146*
2 (SNUBH)	15	44.12	19	46.34	
3 (BRM)	6	17.65	2	4.88	
4 (DH)	8	23.53	7	17.07	
Operative time, min (mean ± SD)	34.4 (± 23.2)		26.8 (± 13.9)		0.100

*Calculated using Fisher’s exact test

### Assessment of the primary end point

**Fig. 3:**
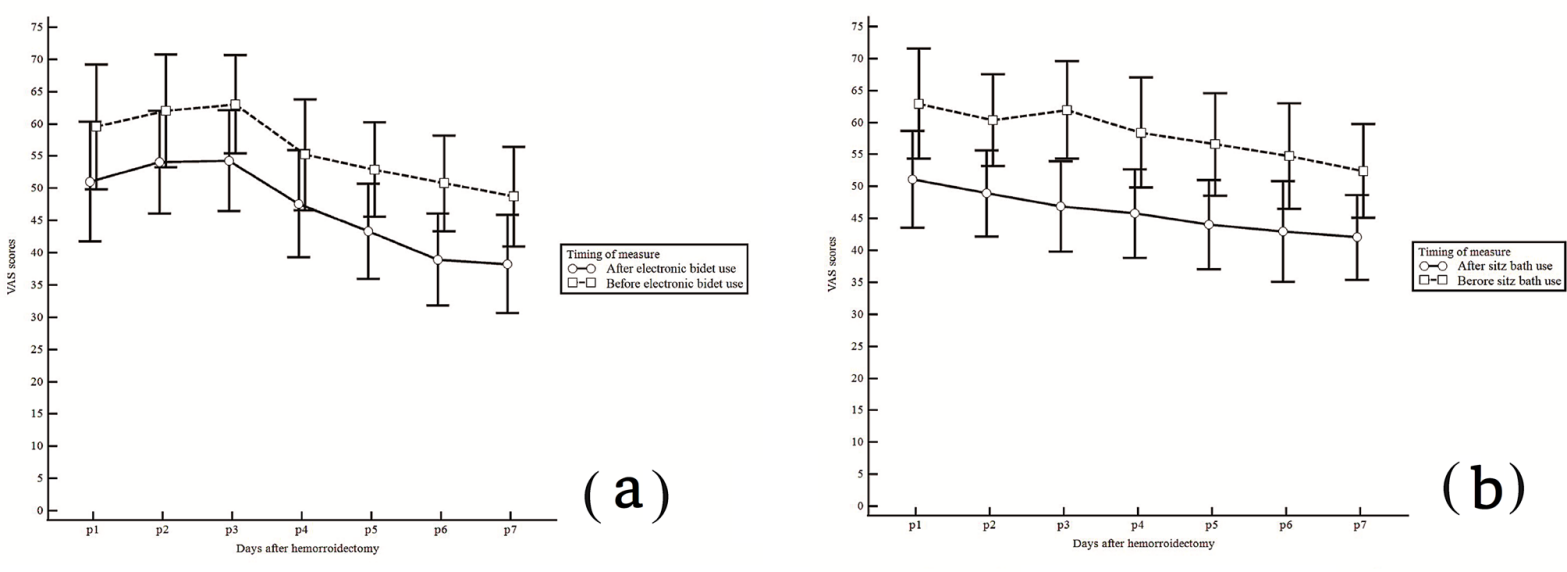
The changes in VAS scores before and after the electronic bidet (**a**) or sitz bath (**b**) for 7 days

**Fig. 4:**
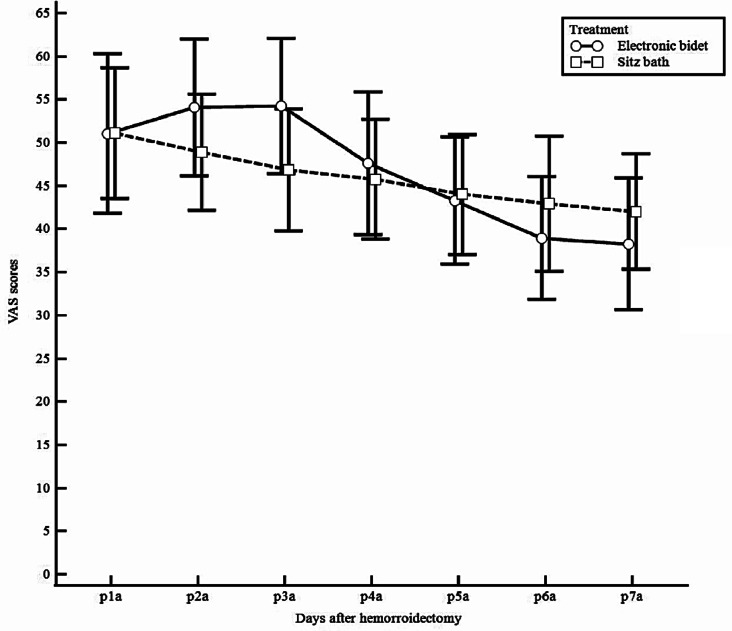
Comparison of VAS scores after electronic bidet and sitz bath for 7 days

The VAS scores for each day of follow-up after hemorrhoidectomy are described in Table 2. The anal pain VAS score after the use of the electronic bidet or sitz bath at 1 week after the operation was not significantly different between the two groups (electronic bidet vs. sitz bath, 38.3 ± 21.9 vs. 42.0 ± 21.1, *p* = 0.453). The upper limit for the 95% confidence interval of the VAS score in the electronic bidet group (81.22) was higher than the margin of noninferiority (46.20), which was predefined as a 10% higher value than the average pain score in the sitz bath group. In the PP analysis, quantitative evidence for noninferiority was not found, with the mean VAS scores of the 2 groups being very similar. The changes in VAS scores before and after the electronic bidet or sitz bath for 7 days are shown in Fig. 3. The difference in VAS scores after only electronic bidet or sitz bath use for 7 days is shown in Fig. 4.

**Table 2:** Visual analog scale score after hemorrhoidectomy

	Before				*p* value	After				*p* value
	Electronic bidet (*n* = 34)		Sitz bath (*n* = 41)			Electronic bidet (*n* = 34)		Sitz bath (*n* = 41)		
	Mean	SD	Mean	SD		Mean	SD	Mean	SD	
POD#1	59.6	27.8	62.9	27.3	0.599	^a^51.1	26.1	51.1	24.0	0.995
POD#2	62.0	25.1	60.4	22.7	0.768	54.1	22.8	48.9	21.4	0.314
POD#3	63.0	21.9	62.0	24.2	0.842	54.3	22.4	^b^46.9	22.1	0.158
POD#4	^a^55.2	24.2	58.4	27.3	0.599	^a^47.6	23.4	45.8	22.0	0.731
POD#5	^a^52.9	20.7	56.6	25.4	0.505	^a^43.3	20.8	44.0	22.0	0.886
POD#6	50.8	21.3	54.8	26.2	0.481	39.0	20.4	42.9	24.9	0.461
POD#7	48.7	22.2	52.4	23.2	0.482	38.3	21.9	42.0	21.1	0.453

^a^, *n* = 33, ^b^, *n* = 40, VAS, visual analog scale, POD, postoperative day, SD, standard deviation

### Assessment of the secondary end point

The wound healing rate at 4 weeks after hemorrhoidectomy was 97.1% (33/34) in the electronic bidet group and 97.6% (40/41) in the sitz bath group (*p* = 0.893). 100% of the participants in both groups completed the patient-reported questionnaire on the convenience of postoperative care at 4 weeks after the operation. Twenty-nine (29/34, 85.3%) participants in the electronic bidet group and 23 (23/41, 56.1%) in the sitz bath group reported that their mode of postoperative care was considerably or completely convenient (*p* = 0.006) (Figure A2).

### Safety assessment

Three patients had serious adverse events during the study period. One patient in the electronic bidet group developed severe headache, and 1 patient in the electronic bidet group and 1 patient in the sitz bath group developed hematochezia. All patients were treated with conservative management, and the problems were resolved successfully. Overall, the postoperative complications were not different between the two groups (*p* = 0.656). Skin tag was the most common complication, affecting 9 patients (25.6%) in the electronic bidet group and 12 (29.3%) in the sitz bath group (*p* = 0.788). The postoperative complications are described in Table 3.

**Table 3:** Postoperative complications

	Bidet (*n* = 34)		Sitz bath (*n* = 41)		*p*-value
	*N*	%	*N*	%	
Complications	15	44.1	16	39.0	0.656
Skin tag	9	26.47	12	29.27	0.788
Anal bleeding	4	11.76	2	4.88	0.401*
Constipation	1	2.94	0	0	0.453*
Diarrhea	0	0	1	2.44	1.000*
Fever	0	0	1	2.44	1.000*
Headache	1	2.94	0	0	0.453*

*Calculated using Fisher’s exact test

## Discussion and conclusion

In this randomized controlled trial, we compared the efficacy and safety of an electronic bidet with a lower-force fountain-type water flow system for anal pain control and wound cleansing after hemorrhoidectomy with those of the conventional warm sitz bath. We could not verify the noninferiority of the electronic bidet, although the VAS score for anal pain 7 days after hemorrhoidectomy did not differ between the two groups.

Due to the considerable variability in VAS scores compared to the previous study that we used as a reference for sample size calculation, we could not demonstrate the noninferiority of the electronic bidet with our data, as the VAS score for anal pain 7 days after hemorrhoidectomy was very similar between the two groups. This might be because the patients in our study presented a much higher level of pain than the subjects in the study that was used as a reference for calculating the sample size [21], and the standard deviation was relatively large. Although the mean overall pain score was approximately 22 in the reference study, the mean pain score after the intervention in the present study was approximately 40. This difference could be due to differences in the study population; the patients in the previous study had acute anal fissures, and our patients underwent hemorrhoidectomy.

Postoperative pain is a concern after hemorrhoidectomy, and many surgeons have tried to find more effective ways to relieve discomfort after anal operations. Sitz baths are commonly recommended postoperatively for cleansing perianal wounds and relieving pain [12–14]. Warm water has been suggested to be most helpful because it enhances wound healing by increasing arterial and venous blood flow and reducing spasms by relaxing the anal sphincter muscle [15, 16]. Neural pathways through a thermosphincteric reflex from perianal skin receptors have been considered to evoke internal sphincter relaxation and pain relief [23]. Local thermal stimulation from a warm sitz bath could relax a hypertonic anal sphincter through a somatoanal reflex and is recommended for use by patients with anal fissures or hemorrhoids as an easy and feasible clinical application [24]. We also found that warm water could decrease the anal resting pressure and relax the anal sphincter [17, 18]. 

Electronic bidets are automatic toilet devices for cleansing the perineal area after defecation and are widely used as household appliances. The electronic system was developed to deliver a water jet to the perineal area and is combined with a conventional toilet seat [17, 25, 26]. The electronic bidet can be used clinically to replace a sitz bath because of the similarity of the contact between the water and the perineal area [25, 26]. We previously studied the effects of electronic bidets on anorectal pressure according to various types and forces of their water streams. Our electronic bidet with a very low-force flow and a fountain-type water stream was developed for clinical use for perianal problems and has been verified to reduce anal resting pressure in a way that is similar to that of warm sitz baths. As gentle initial contact with warm water can prevent the anocutaneous reflex and sphincter muscle spasms, our electronic bidet can be considered for helping to reduce anal pain after anal operations [18]. The secondary endpoint of our study included the results of a comprehensive patient-reported assessment of the convenience of postoperative care and the electronic bidet was more convenient to use for wound management after hemorrhoidectomy. Further research should be conducted to evaluate the acceptability and comfort of different types of sitz baths with the electronic bidet. The findings of this study suggest important implications for postoperative care in hemorrhoidectomy patients. Exploring alternative methods for wound management and pain control, this research highlights the potential of electronic bidets as a convenient and effective alternative to traditional sitz baths, offering a more accessible and user-friendly approach to improving recovery.

This study has some limitations. We performed this study with a small number of patients, which did not enable us to identify all the clinical effects of the electronic bidet. However, this study was a randomized controlled trial and the first study to examine the clinical use of electronic bidets, and it verified their efficacy and safety for treating anal pain and wound management after hemorrhoidectomy. Despite this well-designed study, the small sample size and short-term follow-up results did not allow us to reach a sufficient conclusion, and more studies with a large number of patients and long-term follow-up are necessary. Another limitation is the potential variability in surgical techniques inherent to multicenter studies, as patients were operated on by seven surgeons across four hospitals. Some patients received closed hemorrhoidectomy, while others underwent semi-closed hemorrhoidectomy. We were unable to analyze differences in postoperative pain based on these surgical techniques.

Recent literature has explored innovative approaches to managing postoperative pain following hemorrhoidectomy. A systematic review by Zhang et al. highlighted the potential of acupuncture in relieving post-hemorrhoidectomy pain, suggesting alternative pain management strategies [27]. Colombo et al. conducted an observational study demonstrating the efficacy of mesoglycan in pain control after open excisional hemorrhoidectomy, offering valuable insights into pharmacological interventions [28]. Furthermore, a recent randomized controlled trial by Martínez-Pérez et al. investigated the use of intradermal methylene blue for posthemorrhoidectomy pain relief, presenting another promising approach [29]. These studies reflect the growing array of strategies to address postoperative pain more effectively. Our study contributes to this evolving field by examining a non-pharmacological method for pain management and wound care. Although we could not definitively establish the noninferiority of the electronic bidet, our findings highlight potential benefits that warrant further investigation.

## Conclusions

The VAS score for anal pain 7 days after hemorrhoidectomy did not differ between the electronic bidet and sitz bath groups. However, we could not find quantitative evidence to verify the noninferiority of the electronic bidet with a lower-force fountain-type water flow system for anal pain control after hemorrhoidectomy. Wound healing was similar between the two groups, and the electronic bidet was more convenient to use for wound management after hemorrhoidectomy. Further research is necessary to support the widespread use of electronic bidets for various anal problems.

## Electronic supplementary material

Below is the link to the electronic supplementary material.

Supplementary Material 1

Supplementary Material 2

Supplementary Material 3

Supplementary Material 4

Supplementary Material 5

Supplementary Material 6

Supplementary Material 7

## Data Availability

The datasets used and/or analysed during the current study available from the corresponding author on reasonable request.
