# Supplementary material for: Comparison of warm sitz bath and electronic bidet with a lower-force water flow for postoperative management after hemorrhoidectomy (BIDLOW)
Source: BMC Surg. 2025 Jan 6;25:5. doi: 10.1186/s12893-024-02737-0 (PMC11702218; doi:10.1186/s12893-024-02737-0)
Supplement: Supplementary file 2 — Supplementary Material 2 [file 12893_2024_2737_MOESM2_ESM.docx]

**Patient convenience questionnaire**

1. **Have you ever tried a sitz bath?**

□.Yes □.No

1. **How often do you take a sitz bath?**

□. Almost every day □. 2-3 times a week □. 3-4 times a month □. Less than 2 times a month

1. **Have you ever had any discomfort while taking a sitz bath? If so, please write down everything you can think of.**
2. **For sitz bath group**

**a). How satisfied was the temperature of the sitz bath water you experienced?**

□. Completely unsatisfied □. Considerably unsatisfied □. Neutral

□. Considerably satisfied □. Completely satisfied

**a-1) (Only for unsatisfied respondents) You responded that the temperature was unsatisfactory, why is that?**

□. The temperature is too cold □. The temperature is rather cold □. The temperature is too hot

□. The temperature is rather hot □. Others

**b) Were the devices for a sitz bath convenient? Please rate how convenient the devices for a sitz bath were in taking a sitz bath.**

□. Completely inconvenient □. Considerably inconvenient □. Neutral

□. Considerably convenient □.Completely convenient

**b-1) Why did you respond as above? Please write down what made you uncomfortable**

**c) Considering all the factors you answered above, are you generally satisfied with the sitz bath you are experiencing?**

□. Completely unsatisfied □. Considerably unsatisfied □. Neutral

□. Considerably satisfied □. Completely satisfied

**c-1) Why did you respond as above? Please write down what made you uncomfortable**

**d) In addition, please write down if there were any inconveniences while participating in the test**

1. **For bidet group**

**a) How satisfied was the temperature of the water of the bidet stream you experienced as a sitz bath?**

□. Completely unsatisfied □. Considerably unsatisfied □. Neutral

□. Considerably satisfied □. Completely satisfied

**a-1) (Only for unsatisfied respondents) You responded that the temperature was unsatisfactory, why is that?**

□. The temperature is too cold □. The temperature is rather cold □. The temperature is too hot

□. The temperature is rather hot □. Others

**b) How satisfied was the pressure of the water of the bidet stream you experienced as a sitz bath?**

□. Completely unsatisfied □. Considerably unsatisfied □. Neutral

□. Considerably satisfied □. Completely satisfied

**b-1) (Only for unsatisfied respondents) You responded that the pressure was unsatisfactory, why is that?**

□. The pressure is too strong □. The pressure is rather strong □. The pressure is too weak

□. The pressure is rather weak □. Others

**c) How appropriate was the temperature of the bidet seat to use as a sitz bath?**

□. Completely inconvenient □. Considerably inconvenient □. Neutral

□. Considerably convenient □.Completely convenient

**c-1) Why did you respond as above? Please write down what you felt uncomfortable.**

**d) How convenient was the bidet device you experienced in using the sitz bath function?**

□. Completely inconvenient □. Considerably inconvenient □. Neutral

□. Considerably convenient □.Completely convenient

**d-1) Why did you respond as above? Please write down what you felt uncomfortable.**

**e) Considering the factors you answered above, are you satisfied with the bidet you are currently using as a sitz bath?**

□. Completely unsatisfied □. Considerably unsatisfied □. Neutral

□. Considerably satisfied □. Completely satisfied

**e-1) Why did you respond as above? Please write down what made you uncomfortable**

**f) In addition, please write down if there were any inconveniences while participating in the test**
