# Supplementary material for: Comparison of warm sitz bath and electronic bidet with a lower-force water flow for postoperative management after hemorrhoidectomy (BIDLOW)
Source: BMC Surg. 2025 Jan 6;25:5. doi: 10.1186/s12893-024-02737-0 (PMC11702218; doi:10.1186/s12893-024-02737-0)
Supplement: Supplementary file 3 — Supplementary Material 3 [file 12893_2024_2737_MOESM3_ESM.docx]

**Data Sharing Statement**

**Data**

**Data available:** Yes

**Data types:** Deidentified participant data

**How to access data:** request via a written proposal, email requests to

sbryoomd@gmail.com

**When available:** With publication

**Supporting Documents**

**Document types:** None

**Additional Information**

**Who can access the data:** researchers whose proposed use of the data has been approved

**Types of analyses**: for a specified purpose

**Mechanisms of data availability:** after approval of a proposal and with a signed data sharing agreement

**Any additional restrictions:** individual participant data and the data dictionary will be provided for the purposes of secondary analyses only upon request via a written proposal. Each request will need to include the research question of interest, planned methods, and specify which data points from the trial will be needed for the analysis. The Principal Investigators will review each proposal and decide on whether the secondary analysis is feasible. After a data sharing agreement is finalized, data files will be shared using a secure, online cloud storage infrastructure
