# Supplementary material for: Comparison of warm sitz bath and electronic bidet with a lower-force water flow for postoperative management after hemorrhoidectomy (BIDLOW)
Source: BMC Surg. 2025 Jan 6;25:5. doi: 10.1186/s12893-024-02737-0 (PMC11702218; doi:10.1186/s12893-024-02737-0)
Supplement: Supplementary file 4 — Supplementary Material 4 [file 12893_2024_2737_MOESM4_ESM.doc]

**
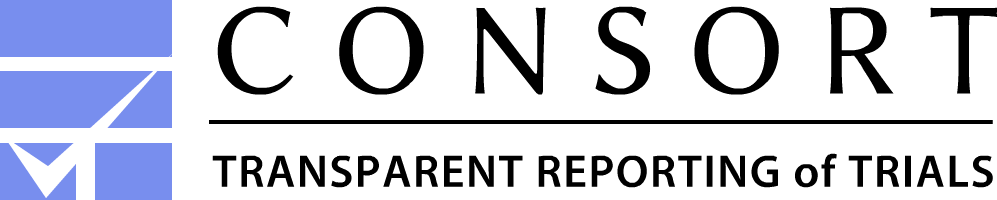
**

**CONSORT 2010 Flow Diagram**

**Allocation**

**Analysis**

**Follow-Up**

**Enrollment**

Assessed for eligibility (n=101)

Excluded (n= 0 )

♦

Analysed (n=33 )
♦ Excluded from analysis (give reasons) (n= 1 ) : low compliance

Lost to follow-up (give reasons) (n= 0 )

Discontinued intervention (give reasons) (n= 0 )

Allocated to intervention **Electronic Bidet (n= 51)**

♦ Received allocated intervention (n=34 )

♦ Did not receive allocated intervention (give reasons) (n=17 )

- Declined study participation (n=12)
- Drug allergy (n=3)
- Unable to install electronic bidet (n=2)

Lost to follow-up (give reasons) (n= 0 )

Discontinued intervention (give reasons) (n= 0)

Allocated to intervention **Sitz Bath** (n= 50 )

♦ Received allocated intervention (n= 41 )

♦ Did not receive allocated intervention (give reasons) (n= 9 )

- Declined study participation (n=7)
- Previous anal disease (n=1)
- Identified a tumor (n=1)

Analysed (n=41 )
♦ Excluded from analysis (give reasons) (n=0 )

Randomized (n= 101)
