# Supplementary material for: Comparison of warm sitz bath and electronic bidet with a lower-force water flow for postoperative management after hemorrhoidectomy (BIDLOW)
Source: BMC Surg. 2025 Jan 6;25:5. doi: 10.1186/s12893-024-02737-0 (PMC11702218; doi:10.1186/s12893-024-02737-0)

**Supplementry legend**

**Table S1.** Demographics and Baseline Characteristics of the Patients in the Per-Protocol Population (n=74)

**Figure S1.** The severity of anal pain after hemorrhoidectomy was assessed by using the visual analog scale (VAS).

**Figure S2.** The results of patient-reported convenience of postoperative care questionnaire which was scored as follows: 1: completely inconvenient, 2: considerably inconvenient, 3: neutral, 4: considerably convenient, and 5: completely convenient.

**Supplemental metarial Table S1. Demographics and Baseline Characteristics of the Patients in the Per-Protocol Population (n=74)**

|  | **Bidet (n=33)** | |  | **Sitz bath (n=41)** | | **p-value** |
| --- | --- | --- | --- | --- | --- | --- |
|  | **N** | **%** |  | **N** | **%** |  |
| Age, years (mean ± SD) | 48.6 (± 11.7) | |  | 52.6+ (± 11.0) | | 0.133 |
| Sex, n (%) |  |  |  |  |  |  |
| Female | 15 | 45.5 |  | 23 | 56.1 | 0.363 |
| Male | 18 | 54.5 |  | 18 | 43.9 |  |
| Hemorrhoid grade |  |  |  |  |  |  |
| II | 1 | 3.0 |  | 2 | 4.9 | 0.394* |
| III | 30 | 90.9 |  | 39 | 95.1 |  |
| IV | 2 | 6.1 |  | 0 | 0 |  |
| Hospitals, n (%) |  |  |  |  |  |  |
| 1 (SNUH) | 4 | 12.1 |  | 13 | 31.7 | 0.094* |
| 2 (SNUBH) | 15 | 45.5 |  | 19 | 46.3 |  |
| 3 (BRM) | 6 | 18.2 |  | 2 | 4.9 |  |
| 4 (DH) | 8 | 24.2 |  | 7 | 17.1 |  |
| Operative time, min (mean ± SD) | 34.2 (± 23.6) | |  | 26.8 (± 13.9) | | 0.116 |

*Calculated using Fisher’s exact test

**Supplemental metarial_Figure S1. The severity of anal pain after hemorrhoidectomy was assessed by using the visual analog scale (VAS).**

**
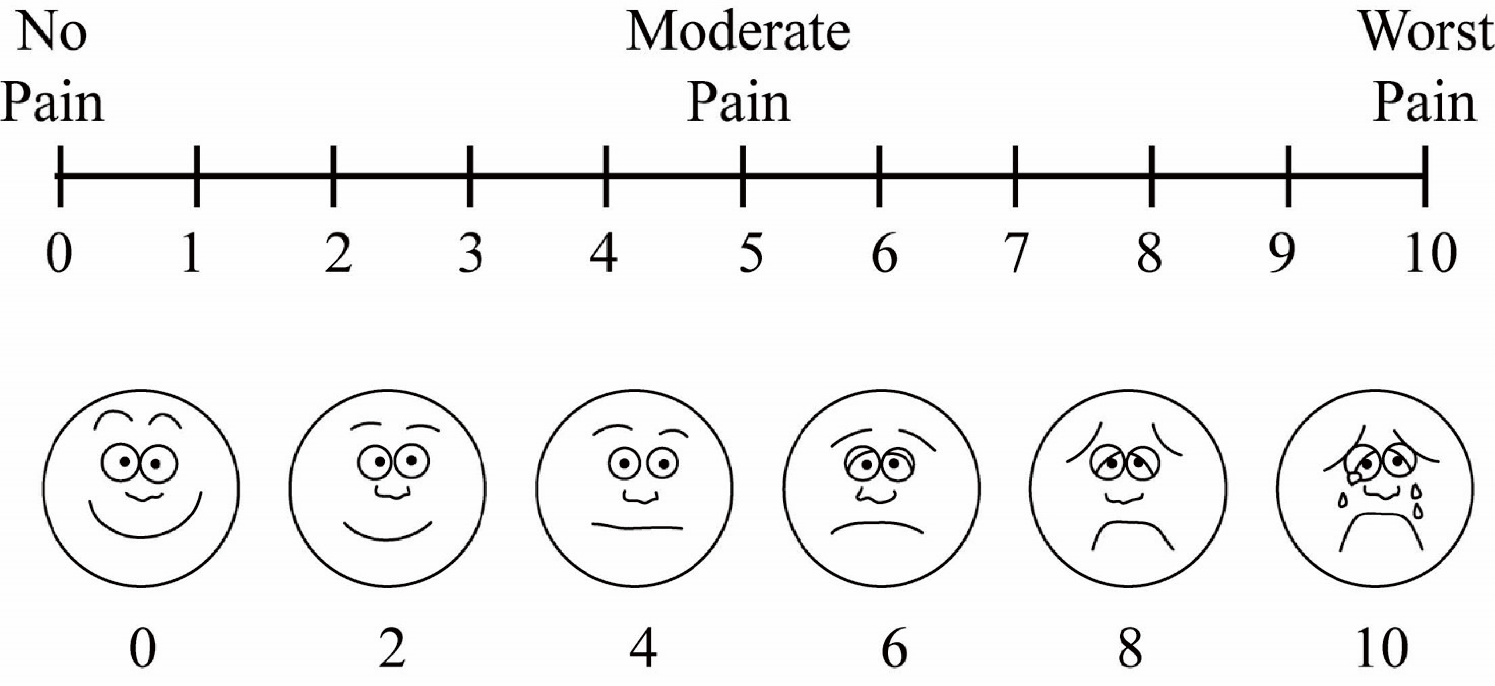
**

**Supplemental metarial_Figure S2. The results of patient-reported convenience of postoperative care questionnaire which was scored as follows: 1: completely inconvenient, 2: considerably inconvenient, 3: neutral, 4: considerably convenient, and 5: completely convenient.**


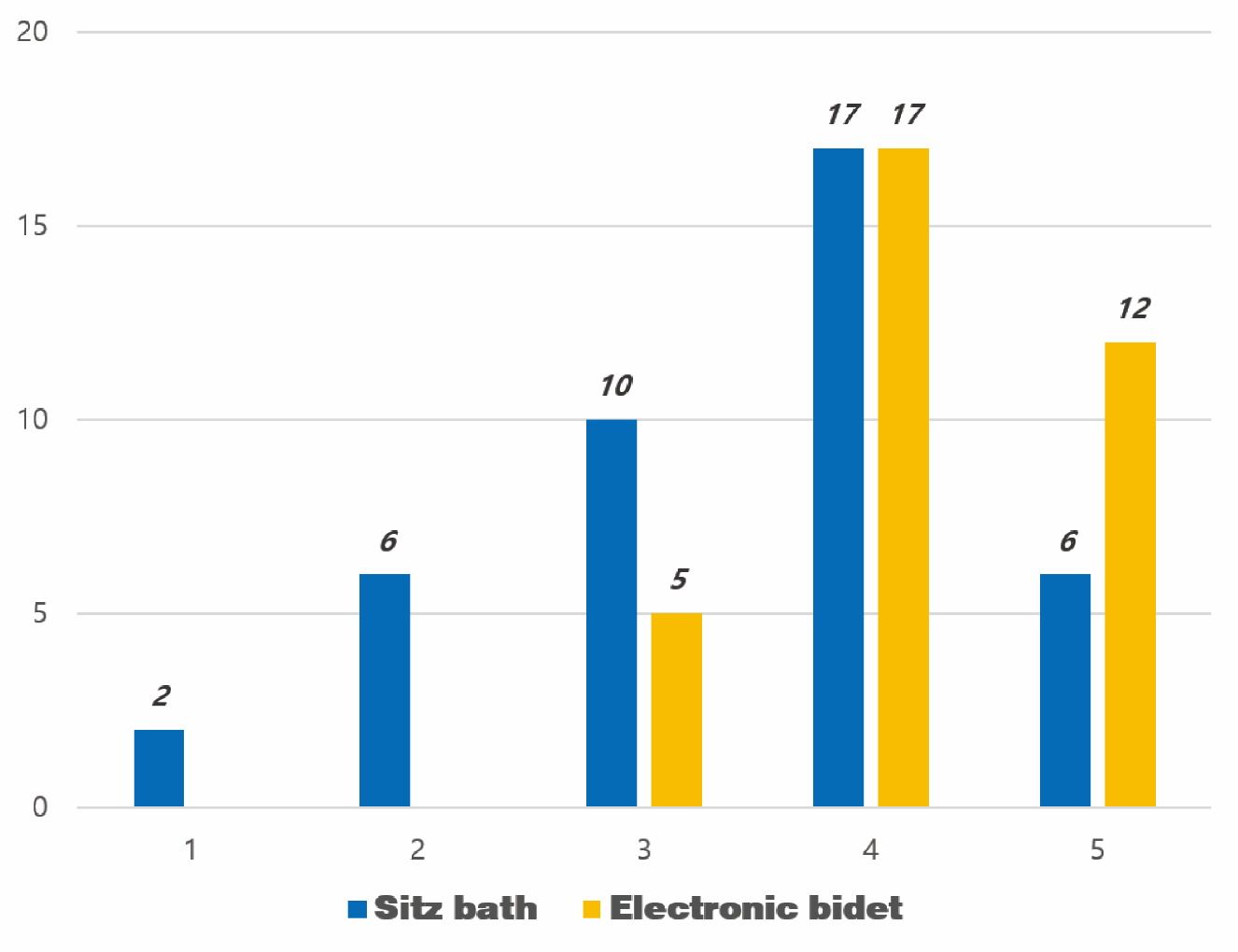

Supplement: Supplementary file 5 — Supplementary Material 5 [file 12893_2024_2737_MOESM5_ESM.docx]
