# Supplementary material for: Comparison of warm sitz bath and electronic bidet with a lower-force water flow for postoperative management after hemorrhoidectomy (BIDLOW)
Source: BMC Surg. 2025 Jan 6;25:5. doi: 10.1186/s12893-024-02737-0 (PMC11702218; doi:10.1186/s12893-024-02737-0)
Supplement: Supplementary file 6 — Supplementary Material 6 [file 12893_2024_2737_MOESM6_ESM.docx]

**Single-Blind, Randomized Controlled, Multicenter Study to Evaluate the Efficacy and Convenience of Electronic Bidet with Very Low Force Flow Fountain System for Pain Control and Wound Healing after Operation for Anal Disease, Compared to Conventional Sitz Bath**

**Version No: 1.7**

**Affiliation of Principal Investigator: Department of Surgery, Seoul National University Hospital**

**Name of Principal Investigator: Kyu Joo Park**

**Summary**

| Title | (Korean) 항문 질환 수술 환자에서 기존 좌욕과 좌욕수류비데 시스템을 이용한 통증 관리 및 창상치유 효과와 편의성 비교를 위한 무작위배정, 다기관 비교 임상시험 |
| --- | --- |
|  | (English) Randomized Controlled, Multicenter Study to Evaluate the Efficacy and Convenience of Electronic Bidet with Low Force Flow Fountain System for Pain Control and Wound Healing after Operation for Anal Disease, Compared to Conventional Sitz Bath |
| Principal Investigator | Department of Surgery, Seoul National University College of Medicine, Professor Kyu Joo Park |
| Research Funding Organization | COWAY CO., LTD., Seoul National University College of Medicine Research Foundation |

| Study Objective | After operation for anal disease, sitz bathing is recommended for pain control and wound healing. However, the process of filling with water and draining of the conventional sitz bath is very cumbersome and difficult, so it is not easy to do it for patients with discomfort to move due to severe pain after surgery.  Like a sitz bath, bidet brings water into contact with the perianal area. Because of similarities between the sitz bath and the bidet, questions have been constantly raised whether a bidet could be used instead of a sitz bath. But, these effects of the bidet have not been studied up to date. Accordingly, we reported that bidets can have the same effect as sitz baths in reducing anorectal pressure in previous studies.  However, clinical studies comparing the effects of sitz baths and bidets are necessary to be applied to the treatment of patients in clinical practice. In this study, we tried to compare and analyze the efficacy and convenience for pain control and wound healing after operation for anal diseases using a bidet with a very low force water stream compared to the conventional sitz bath. |
| --- | --- |
| Study Design | Randomized Controlled, Multicenter Clinical Trial  (non-inferiority trial) |
| Study Duration | 2015.01.12 ~ 2019.01.11 |
| Subject of Study (test drug, etc.) | Patients who underwent a hemorrhoidectomy |
| Number of Subjects | 95 subjects |
| Vulnerable Subjects | (N/A) |
| Study Method | 1. Patients for whom the operation for the anal disease has been determined in the outpatient clinic are divided into a sitz bath group and bidet group through randomization prior to the surgery. This is a single-blind study and the investigator is unaware of which group the patients were assigned to. 2. From the next day after the operation for anal disease, perform a sitz bath or bidet at least once a day. It must be performed in the morning and can be additionally performed if there is a pain in the middle. 3. The pain is assessed before and after the use of a sitz bath or bidet for 4 weeks after surgery. The degree of pain is evaluated by a subjective Visual Analog Scale (VAS). Time to use a sitz bath or bidet is recorded. 4. Every week after surgery, the outpatient clinic visit is made to evaluate the healing of anal wounds. Complete wound healing requires that the wound area is epithelialized and there is no exudate. 5. As the primary endpoint, the difference in pain is compared and analyzed at 1 week. 6. As a secondary endpoint, a questionnaire survey for the difference in wound healing and the convenience of sitz baths and bidets after 4 weeks is conducted to analyze and compare. 7. As a secondary endpoint, evaluating the urinary function before and after surgery. |
| Efficacy Evaluation | 1. Primary endpoint - the degree of pain (VAS) after operation for anal disease 2. Secondary endpoints - wound healing and patient convenience, urinary function before and after surgery in men over 40 years old |
| Safety Evaluation | After the anal surgery, visit an outpatient clinic to perform anal visual inspection and palpation. Through this, adverse events or side effects that may occur due to bidet or sitz baths will be evaluated, and examinations are conducted considering the possibility of anal fissure, rectal prolapse, perianal burns. If an adverse event or side effect occurs, document and report it to the IRB, and report any serious adverse event (SAE) immediately. |
| Expected Effect and Predicted Outcome | The sitz bath is widely used in the treatment of anal diseases, but it is an undeniable fact that it is a very difficult treatment process for patients after surgery. The use of a bidet after anal surgery is not inferior to the use of a sitz bath in pain management and wound healing. It is thought that a bidet can be a substitute for a sitz bath if it is more convenient, and it can be used for pain and wound management after anal surgery as a more favorable method. |

**Study Protocol**

1. **Study Title**

항문 질환 수술 환자에서 기존 좌욕과 좌욕수류비데 시스템을 이용한 통증 관리 및 창상치유 효과와 편의성 비교를 위한 다기관, 무작위배정, 비교 임상시험

(Phase II, Randomized Controlled, Multicenter Study to Evaluate the Efficacy and Convenience of Electronic Bidet with Low Force Flow Fountain System for Pain Control and Wound Healing after Operation for Anal Disease, Compared to Conventional Sitz Bath)

1. **Name and Address of the Study Institution**
2. Department of Surgery, Seoul National University College of Medicine

(101 Daehangno (28 Yongon‐dong), Jongno‐Gu, Seoul)

1. Daehang Hospital

(2151 Nambusunhwan-ro (481-10 Bangbae 3-dong), Seocho-gu, Seoul)

1. **Name and Title of Principal Investigator and Co-Investigators**
2. **Principal Investigator**

Kyu Joo Park, Department of Surgery, Seoul National University College of Medicine, Professor

1. **Co-Investigators**

Seung-Bum Ryoo, Department of Surgery, Seoul National University College of Medicine, Clinical Assistant Professor

Jae Beom Lee, Daehang Hospital, Manager

Ki Ho Song, Daehang Hospital, Manager

Jung Hyung Joong, Daehang Hospital, Manager

Kim, Duck-Woo, Department of Surgery, Seoul National University College of Medicine (Bundang Hospital), Clinical Associate Professor

Heung-Kwon Oh, Department of Surgery, Seoul National University College of Medicine(Bundang Hospital), Clinical Associate Professor

Seung Chul Heo, Department of Surgery, Seoul National University College of Medicine(Borame Medical Center), Associate Professor

Ru Mi Shin, Department of Surgery, Seoul National University College of Medicine(Borame Medical Center), Clinical Assistant Professor

1. **Sub-Investigators**

Yoon-Hye Kwon, Department of Surgery, Seoul National University College of Medicine, Fellow

Dong Woon Lee, Department of Surgery, Seoul National University College of Medicine, Fellow

In Ho Song, Department of Surgery, Seoul National University College of Medicine, Fellow

Myeong-Ja Kang, Department of Surgery, Seoul National University College of Medicine, Clinical Research Nurse

Hoon Sung, Daehang Hospital, Clinical Research Nurse

Eun Hwa Lee, Daehang Hospital, Clinical Research Nurse

Jin Ah Kim, Department of Surgery, Seoul National University College of Medicine (Bundang Hospital), Clinical Research Nurse

Byung-Hoon Son, Department of Surgery, Seoul National University College of Medicine (Borame Medical Center), Fellow

1. **Sponsor**N/A
2. **Name and Address of Research Funding Organization**

COWAY CO., LTD.

(56-39 Nakseongdae-ro 15-gil, Bongcheon-dongGwanak-gu, Seoul, South Korea)

Seoul National University College of Medicine Medical Education Research Foundation Designated Fund for Laboratory

1. **Estimated Study Duration**

2015.01.12 - 2019.01.11

1. **Indications to be studied**

the patients who undergo hemorrhoidectomy

1. **Study Background and Objectives**
2. **Study Background**

The sitz bath is usually recommended for hygiene and also as a therapeutic modality for the treatment of anorectal diseases. The patient seats on the basin or tub, and sinks their buttocks into the water, especially the perianal area, for a few minutes. In particular, a warm sitz bath could be helpful to reduce pains by reducing the anal pressure through relaxing the muscle and relieve congestion and edema by increasing the blood ﬂow to the perianal area. The effect of such a warm sitz bath has been proven through many studies for a long time, and it is essential for pain control and wound healing after operation for anal diseases [1-3].

A bidet is mainly used for cleansing the anal and perineal area [4-5]. Because it brings water in contact with the perianal area, similar to a sitz bath, many patients actually use a bidet at home, although there are few reports on its clinical use.

In previous studies, we have already reported that using a bidet in normal adults can effectively lower the anorectal pressure when using low water pressure and warm water [6]. It has been reported that if this effect of reducing the anorectal pressure is lower than that of the conventional commercial bidet, it can have the same effect as a sitz bath with a decrease in the initial anal pressure [7, unpublished].

Relieving pain after surgery is mainly performed by using analgesics, so sitz baths and bidets are limited to play a subsidiary role. However, it is thought that it can be helpful if it can be used conveniently. However, to use a bidet with the lowest force water stream as a substitute for a sitz bath for pain control and wound healing after anal surgery for patients in clinical practice, a randomized comparative clinical trial is necessary. In addition, since a sitz bath is helpful for patients who have urinary difficulty after surgery, we would like to compare it as a secondary endpoint whether the bidet is also effective for this.a4

1. **Study Hypothesis and Objective**

This study is intended to prove that, as a primary endpoint, the degree of pain in the case of using a bidet after operation for the anal disease is not inferior to compared to the conventional sitz bath. Also, as a secondary endpoint, we tried to demonstrate that there is no difference in wound healing within 4 weeks and the patient's convenience can be increased.

Therefore, in this study, we tried to compare and analyze the effect of pain control and wound healing after operation for anal diseases, the convenience, and urinary difficulty improvement with the conventional sitz bath by using a bidet with the lowest force water stream.

This trial is a study to compare and analyze whether a general home appliance bidet can replace the sitz bath therapy (using a sitz bath basin), which is generally used for pain control and wound management of patients after anal surgery. This is a comparative analytical study on the equivalent effect between sitz bath therapy using a sitz bath basin, not medical devices, and a bidet. It is not a clinical trial for the medical device.

1. **Code Name of Investigational Product or Investigational Device (or generic name of the active ingredient), Drug ingredients and quantities, Formulation, etc. (including control drug)**

N/A

(Bidet - Coway Clinic Bidet (BASH21-A) is used

It is a product that is currently marketed as a home appliance and is not a medical device. Instructions/manual is attached.)

1. **Inclusion Criteria and Exclusion Criteria, Target Number of Subjects and Rationales for Calculation**
2. **Inclusion Criteria**
3. 19 to 70 years old
4. Patients who need to undergo hemorrhoidectomy for hemorrhoids
5. Patients who are capable of understanding and conducting this clinical trial
6. **Exclusion Criteria**
7. Patients who have a previous history of anal surgery
8. Patients who underwent PPH for hemorrhoids
9. Crohn's anal disease
10. Complex anal fistula
11. Patients with uncontrollable medical diseases such as high blood pressure or diabetes
12. Active TB patients and patients taking TB drugs
13. HIV patients
14. Patients with a history of malignant tumors within the last 5 years
15. Patients receiving immunosuppressants
16. Pregnant or breastfeeding women
17. Patients with drug allergy
18. **Target Number of Subject and Rationale for Calculation**
19. Non-inferiority test

Reference 14 shows data that the warm water spray method could expect similar effects to the sitz baths. The bidet used in this study is assumed to be non-inferior to the sitz bath in improving post-operative pain. It is assumed that the average of the bidet group and the average of the sitz bath group were the same and the difference was 0.

On the other hand, in Reference 15, in post-OP pain of acute anal fissure, sitz baths showed a tendency to significantly reduce pain. Considering that pain decreases over time and almost disappears at 4 weeks, it can be said that it is a clinically valid approach to show that a bidet is not inferior to a sitz bath on the pain at 1 week. In the references, the average pain of the sitz bath group at 1 week was 22 and the standard deviation was 3.2 for the comparison data of overall pain in Figure 2. In the non-sitz bath group, the average pain was 29 and the standard deviation was 4.5. We try to obtain the number of subjects by substituting this value into the formula for calculating the sample size for the non-inferiority trial.


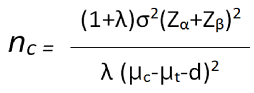


When α=0.025 and β=80%, Zα = 1.96 and Zβ = 0.85. When the non-inferiority margin, delta = 10%, the non-inferiority margin is 2.2, which is 0.1 times the average of 22 in the sitz bath group. That is, the upper limit of 95% of the distribution should not exceed 24.2 in the control group to prove non-inferiority. In the equation, σ is the standard deviation and λ is the non-centrality parameter. In this study, λ=1.

For the standard deviation, 3.2, which is the standard deviation value in the sitz bath group, is used.

The calculation result of the above equation is 34 subjects. Therefore, patient recruitment is continued until the size of FAS, the analysis set of the primary endpoint, reaches a total of 68 subjects (up to 34 per group).

1. **Recruitment Plan of Study Subjects**

For patients who visit the outpatient clinic for anal disease and are judged that anal surgery is necessary, the medical staff will explain this study and obtain consent to recruit study subjects. The principal investigator and sponsor of this study will not exclude patients who are possible to participate in this study based solely on race or socioeconomic status. All efforts will be made to ensure that patients who are eligible for the inclusion criteria can participate in this study, and will notify the objective of this study to patients so that they will represent the entire population of patients being treated at this institution.

Researchers Myeong-Ja Kang and Hwa-Sook Shin, and professor Seung-Bum Ryoo will be in charge of some parts of the study, such as recruitment of study subjects, consent process, data analysis, and preparation of the report. The overall monitoring will be performed by professor Ji-Won Park, who is not related to this study.

1. **Study Method**
2. **Detailed Study Method**
3. Patients for whom the operation for the anal disease has been determined in the outpatient clinic are divided into a sitz bath group and bidet group through randomization prior to the surgery. This is a single-blind study so that the investigator (operator) does not allow the patient to know which group the patient is allocated to.
4. From the next day after the operation for anal disease, perform a sitz bath or bidet at least once a day. It must be performed in the morning and can be additionally performed if there is a pain in the middle.
5. The pain is assessed before and after the use of a sitz bath or bidet for 4 weeks after surgery. The degree of pain is evaluated by a subjective Visual Analog Scale (VAS). Time to use a sitz bath or bidet is recorded.
6. Every week after surgery, the outpatient clinic visit is made to evaluate the healing of anal wounds. Complete wound healing requires that the wound area is epithelialized and there is no exudate.

In the outpatient clinic visit, the investigator should not ask the patient whether to use a sitz bath or bidet.

1. As the primary endpoint, the difference in pain is compared and analyzed at 1 week.
2. As a secondary endpoint, a questionnaire survey for the difference in wound healing and the convenience of sitz baths and bidets after 4 weeks is conducted to analyze and compare.
3. In addition, in men over 40 years of age, evaluating urinary function through a validated questionnaire before and after surgery (at the outpatient visit), respectively.

* CONSORT diagram

Randomization 🡪 bidet group, sitz bath group

(수술 전 외래 방문시)

Operation for anal disease

; Hemorrhoidectomy

**Bidet group**

Use of bidet from the day after surgery

; warm water, low water pressure (less than 4 gf)

(Install the same bidet in the hospital and at home)

**Sitz bath group**

Warm sitz bath from the day after surgery

**Comparative Evaluation of Efficacy**

**1) Pain (scoring)**

**2) Wound healing period (outpatient follow-up)**

**3) Patient convenience (questionaire)**

* Sitz Bathing Method

1. Urinate in advance.

2. Fill with the warm water of 38-40°C in half of the sitz bath basin.

(The water temperature should be warm when soaking the elbow, and if there is too much water, the water will overflow when to sit your buttocks in a sitz bath basin.)

3. Check the front and back of the sitz bath basin and place it on the toilet.

4. Soak anus in a sitz bath basin and sit for 3-5 minutes.

5. After sitz bathing, gently wipe dry around the anus with a towel. In some cases, you can also use a dryer. (It is important to dry thoroughly)


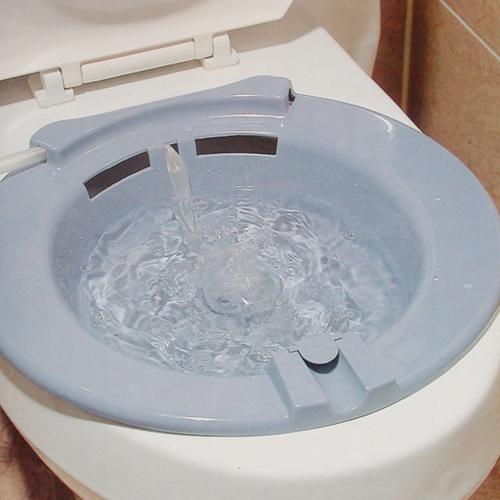


* Bidet Method

1) Sit on the bidet with the lowest water pressure.

2) Press the sitz bath button to bring water flow into contact with the anal wound area for about 3 to 5 minutes.

3) Dry off the anal area.


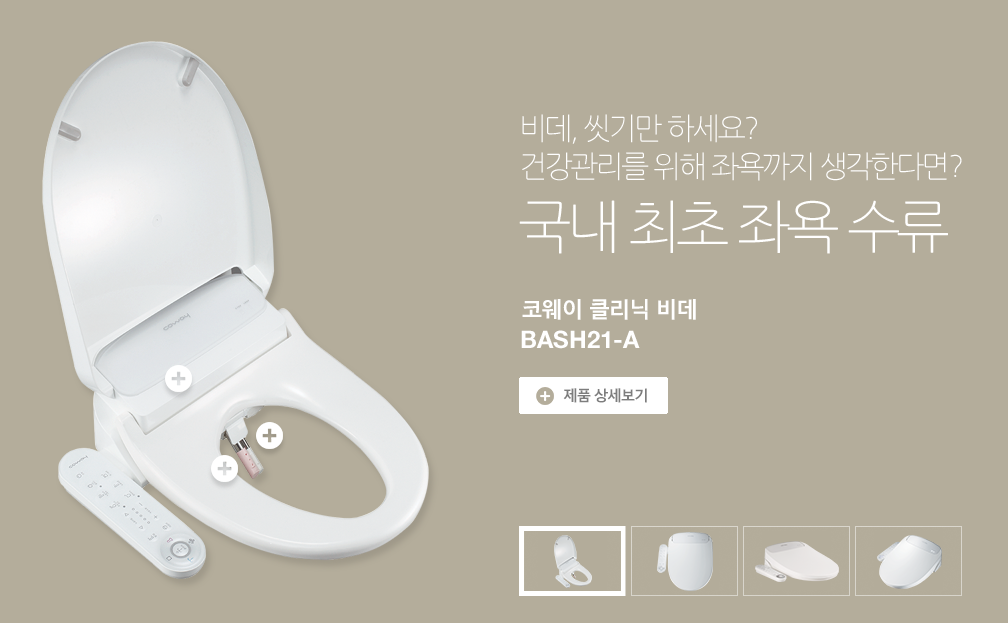


1. **Control Group Setting and Randomization Method**

Randomization is performed using the sitz bath group as a control group. By requesting the Seoul National University Medical Research Collaborating Center (MRCC), a randomization table is created in advance. On the MRCC home page, a web-based randomization system is used to check the patient allocation group the day before surgery and conduct patient education.

1. **Study Drug Administration Dosage, Method of Administration, Combination Therapy, Reasons for Selection when using Control Drug**

Ketorolac tromethamine

It is a general analgesic used for pain in the wound area after surgery, iv 30mg prn (90mg/day) is used on the day of surgery and until the morning of the next day, and 1T (10mg) tid (prn qid) is used for 5 days from the afternoon of the next day of surgery. (However, when the hospitalization period is different depending on the hospital, a PCA device using ketorolac (90mg/day) can be used during the hospitalization period of 3 to 4 days. An oral analgesic is prescribed at the time of discharge)

At the outpatient visit 7 days after surgery, the remaining analgesics will be collected and recorded the amount.

1. **Observation Items, Clinical Laboratory Items, and Observation Test Methods**
2. Primary endpoint – the degree of pain after operation for anal disease
3. Secondary endpoint - wound healing and convenience of performance
4. Secondary endpoint- questionnaires on the degree of urinary difficulty improvement (IPSS), men over 40 years old (subgroup analysis)
5. **Efficacy Evaluation Criteria, Evaluation Method**
6. Primary endpoint evaluation method – The degree of subjective pain is evaluated by the Visual Analog Scale (VAS). The patient records the degree of pain after taking a sitz bath or bidet at a certain time every day (right after waking, 7-8 am). After a week, the degree of pain (after using a sitz bath or bidet immediately after waking up) is used as the primary endpoint for comparative analysis.
7. Secondary endpoint evaluation method- Also, the degree of pain reduction is performed the linear comparative analysis as a continuous variable.
8. Secondary endpoint evaluation method - Every week after anal surgery, the outpatient clinic visit is made to get an anal examination. The physician in the outpatient clinic should confirm complete epithelialization and no exudate from the anal wound through visual inspection and palpation, and it is determined as a complete healing.
9. Secondary endpoint evaluation method – Convenience in performing sitz baths and bidets is investigated through a questionnaire (attached) at 4 weeks after surgery.
10. Secondary endpoint evaluation method – For men over 40 years old, a questionnaire on urinary difficulty is completed and evaluated when visiting the outpatient clinic every week.
11. **Differences from Existing Treatments and Researches**

Conventional sitz baths performed after anal surgery is a very cumbersome and difficult process to ﬁll a small tub with water, place it on a toilet, and drain the water after taking sitz baths, so it is a very difficult procedure for patients to perform for 3 to 4 times a day. Therefore, the elderly or disabled with limited mobility cannot do it without the help of a guardian. Like a sitz bath, bidet brings water into contact with the perianal area. Because of similarities between the sitz bath and the bidet, questions are raised whether a bidet can be used instead of a sitz bath. The investigator suggested that bidets can have the same effect as sitz baths in previous studies. If the use of such a bidet has a similar effect to the sitz bath after anal surgery, the patient who has undergone anal surgery can conveniently use a bidet instead of a sitz bath. These effects of the bidet have not been studied up to date, so it is expected that this study can compare and analyze the pain relief effect between bidets and sitz baths after anal surgery.

1. **Benefits and Risks to Study Subjects
   *** **Possible Side Effects/Risks and Countermeasures**

Anal fissure, rectal prolapse, and perianal burns have been reported as adverse events or side effects from the use of a bidet. Most of these cases are high-risk patients with abnormalities in the perianal nerves due to long-term use of the bidet, old age, or diabetes, or it is caused by using water above 50°C [10-12].

Side effects of sitz baths can also be similar to bidets, but the possibility is very low. Therefore, these side effects occur very rarely, but safety evaluation will be made through an outpatient interview or anal examination during clinical trials. When side effects occur, immediate treatment will be performed, and the appropriate treatment cost for this will be supported by the study.

In addition, side effects from anal surgery may occur, which may vary from pain in the wound to simple infections, abscesses, anal fistulas, fecal incontinence, and urinary retention. This will also be evaluated through an outpatient examination, and immediate treatment will be provided when it occurs. However, treatment costs for this are not supported.

Pain that occurs after anal surgery can be significantly reduced with a sitz bath or bidet. For additional pain control, this study planned to use the amount of analgesics commonly used after surgery. However, if the pain is not controlled by the use of these analgesics, it is thought that the occurrence of complications should be suspected. It is considered reasonable to identify complications through outpatient care and decide whether to continue the clinical trial. If the pain persists despite appropriate treatment, it is thought that the dropout of the patient should be considered.

***** **A description of the rationale (or expected scientific/medical benefit) that the study should be conducted despite the predicted side effects/risk**

The sitz bath is widely used in the treatment of anal diseases, but it is an undeniable fact that it is a very difficult treatment process for patients with discomfort after surgery. If a bidet can be a substitute for a sitz bath, it will be a more convenient way after anal surgery as an auxiliary method. To this end, it can be said that a study comparing the pain control and wound healing effects between sitz bath and bidet in patients after surgery is essential.

1. **Criteria for Discontinuation and Dropout**
2. When side effects occur due to anal surgery
3. In case of side effects of sitz bath or bidet
4. When another serious disease occurs during clinical trial and treatment is necessary
5. Others, If clinical trials cannot be performed properly
6. When the subject withdraws the consent of participating in the clinical trial
7. **Safety Evaluation Criteria including Side Effects, Evaluation Method, and Reporting Method**

After anal surgery, an outpatient visit is performed every week for visual anal inspection and palpation. Through this, the adverse events or side effects that may occur due to the bidet or sitz bath are evaluated, and the examination is conducted considering the possibility of anal fissure, rectal prolapse, and perianal burns.

If an adverse event or side effect occurs, document and report it to the IRB, and report any serious adverse event (SAE) immediately. A serious adverse event is defined as any unintended medical phenomenon that occurs in a subject during a clinical trial and is as follows.

1. If it causes death.
2. If it is life-threatening: The meaning of life-threatening refers to the case where the investigator determines that the patient is at risk of death at the time of the event, and it does not include adverse events that may lead to death if it becomes more serious.
3. If hospitalization or prolongation of the hospitalization period is required
4. If permanent or serious disability and dysfunction were induced
5. If congenital anomalies or birth defect in the offspring of the study subjects (male or female) who received the investigational product
6. Reactions that jeopardized subjects or that require medical or surgical treatment to prevent the results listed above, even if they are not included in the criteria listed above for the seriousness.
7. **Data Safety Monitoring Plan (DSMP)**

The person in charge of monitoring (department of surgery, professor Park Ji-won) should regularly (once/year) confirm that the protocol and GCP are being complied with and review the source data to verify that the information in the CRF is accurate. The patient's handwritten VAS and CRF should be confirmed to be consistent.

Also, in conducting this clinical trial, the subjects' evaluation results will be continuously monitored by the principal investigator and safety monitoring will be performed with the help of MRCC in case of a problem. If an adverse event is reported, an immediate report will be made to the IRB and the continuation of the clinical trial will be determined according to the severity of the adverse event.

Recruitment of study subjects, consent process, data analysis, and report preparation for this study will be conducted under the supervision of the person in charge of monitoring who has no conflict of interest with this study.

1. **Data Analysis and Statistical Analysis Method**

Data and statistical analysis will be conducted with the help of MRCC.

Establishment of analysis set: Data analysis is performed by dividing the full analysis set (FAS) and the PP set.

According to ICH E9, FAS is an analysis set that is as close as possible to the concept of the intention-to-treat (ITT) principle and it can exclude the least number of subjects from the analysis for justifiable reasons from all randomized subjects. The reasons for excluding a subject from the analysis target group without the possibility of introducing bias are under the following circumstances.

① Subjects who violate the criteria for inclusion/exclusion of subjects

② Subjects who have never received the study drug

③ Subjects with no data after randomization

In the case of ② and ③, the excluded subjects were due to dropout caused by schedule adjustments with the visiting technician during the installation of a bidet after surgery, and it is expected that there is no relation to the treatment effect. However, to determine whether there are potential biases that may occur by excluding them, we will check whether the underlying characteristics collected during the screening process differ from those included in the analysis.

The PP set consists of subjects who performed sitz baths or bidets as assigned to at least 80% of the set schedule.

The primary endpoint is determined by the FAS.

Analysis of the underlying characteristics and questionnaire of the two groups: Continuous variables are presented using t-test and categorical variables are compared using the chi-squared test. Pain scores are compared for each week using a t-test. Bonferroni correction is implemented in consideration of the expansion of Type I error due to multiple comparisons. For the pain score in the first week after surgery, it is confirmed that the upper limit of 95% of the bidet group is less than the value 10% higher than the average value of the sitz bath group.

Analysis of adverse events and side effects: Identify the frequency of each side effect and check whether there is a significant difference between the two groups through the Fisher test. Analysis of side effects will be performed on all subjects who have conducted a bidet or sitz bath even once.

A statistical test is conducted in a two-tailed test and a p-value less than 0.05 is considered significant.

1. **Study Schedule**

|  | December | January | February | March | April | May | June | July | August | September | October | November |
| --- | --- | --- | --- | --- | --- | --- | --- | --- | --- | --- | --- | --- |
| Building a network of participating hospitals |  |  |  |  |  |  |  |  |  |  |  |  |
| Recruitment of subjects |  |  |  |  |  |  |  |  |  |  |  |  |
| Clinical trial |  |  |  |  |  |  |  |  |  |  |  |  |
| Results analysis and interim reporting |  |  |  |  |  |  |  |  |  |  |  |  |
| Preparation of paper |  |  |  |  |  |  |  |  |  |  |  |  |
| Paper presentation and result report |  |  |  |  |  |  |  |  |  |  |  |  |

1. **Measures for the Safety Protection of the Subjects**
2. **Basic Principal for Ensuring Research Ethics**

This study will comply with the Helsinki Declaration and ICH-GCP revised in 2013 and will be conducted following the protocol approved by the Institutional Review Board (IRB). The purpose and content of the study are explained to the subjects, and only applicants who know and consent to the purpose of this study will participate in the study. Participants in the study can discontinue the trial at any time during the trial and also have been aware of this. There are no special disadvantages to participants related to this study, and the confidentiality of medical information will be maintained. Documents containing the patient's personal information will be retained by encrypting and enclosing in the office of the professor in charge. Access is prohibited except for the investigators involved in this study.

1. **Subjects’ Consent Process**

Before surgery, the medical staff will explain the risks and benefits of this study in the ambulatory care of the department of surgery, and then obtain consent to participate in the study. The principal investigator or sub-Investigators obtains informed consent, and only adult patients who are able to understand and perform this clinical trial are included. Consent can be obtained immediately after explaining the study, and if necessary, it can be obtained at an outpatient visit right before surgery, or when hospitalized for surgery. After sufficient explanation of the study, allow patients to decide by themselves to consent. The investigator explains to the patient in easy words, and the consent form is also explained in an easy-to-understand manner.

1. **Compensation Plan for Subjects**

The subject will receive an amount of 50,000 won as compensation per outpatient visit after surgery, and the initial participation fee (100,000 won) will be supported by the research fund. It is not obliged to participate in the study until the end, but the compensation will be paid differentially if the final termination is not made (total amount of 300,000 won upon completion, but 500,000 won for Daehang Hospital). Also, in the event of side effects caused by a bidet or sitz bath, the appropriate treatment cost will be borne by the investigator.

Study subjects will receive free support for bidet installation or sitz bath.

1. **Confidentiality of Subjects’ Personal Information**

1) The name of the subject is anonymized with initials, and the subject identification and record number are encrypted to be managed by the principal investigator in a separate file so that personal information is not exposed.

2) The use of results or providing them to others for purposes other than the original purpose of this study is limited only if the subject consent in writing. At this time, whether personal information is included depends on the consent of the subject.

3) If the subjects or their legal representative of this study applies requests to access or issue a copy of test results, records regarding the objections, the informed consent form, it should be responded to.

4) In this study, files containing the subject's personal information and examination results will be encrypted and retained, which will only be accessible to the investigator and co-investigators.

5) Samples will be managed and handled by assigning a study participation number that cannot identify individuals.

6) Under Article 15 of the Enforcement Regulations of the Bioethics and Safety Act, records related to the study are kept for 3 years from the end of the study, and documents that are past the retention period are destroyed according to Article 16 of the Enforcement Decree of the Personal Information Protection Act.

1. **Additional protective measures in case of including vulnerable subjects**

(N/A)
